# Evaluation of Normal Thyroid Tissue and Autoimmune Thyroiditis in Children Using Shear Wave Elastography

**DOI:** 10.4274/jcrpe.galenos.2018.2018.0137

**Published:** 2019-05-28

**Authors:** Figen Bakırtaş Palabıyık, Ercan İnci, Esra Deniz Papatya Çakır, Elif Hocaoğlu

**Affiliations:** 1 University of Health Sciences, Bakırköy Dr. Sadi Konuk Training and Research Hospital, Clinic of Radiology, İstanbul, Turkey; 2 University of Health Sciences, Bakırköy Dr. Sadi Konuk Training and Research Hospital, Clinic of Radiology, Division of Pediatrics, İstanbul, Turkey

**Keywords:** Shear wave elastography, children, thyroid, autoimmune thyroiditis

## Abstract

**Objective:**

Shear wave elastography (SWE) is a user-independent ultrasonographic technique that evaluates tissue elasticity. It is used especially in the evaluation of thyroiditis and thyroid nodules when it is capable of distinguishing malignant from benign thyroiditis in adults. To date, no studies have evaluated SWE in pediatric thyroid patients. The aim of this study was to measure the elasticity of normal thyroid tissue in children and adolescents using SWE and to investigate its role in the diagnosis of pediatric autoimmune thyroiditis.

**Methods:**

In total, 113 healthy children of whom 66 (58.4%) were girls and 57 children with autoimmune thyroiditis of whom 45 (78.9%) were girls were evaluated by SWE after B-mode ultrasound. The quantitative evaluation of normal thyroid tissue in healthy children and those with autoimmune thyroiditis was performed using shear wave velocity (SWV) values (m/s). Thyroid antibodies were consistent with autoimmune thyroiditis. Data were compared using descriptive and analytical statistics and receiver-operating characteristic curves.

**Results:**

The mean ± standard deviation (range) of SWV value in thyroid parenchyma of the healthy children was 1.82±0.3 m/s (1.32-2.37) m/s. There was a significant positive correlation between age and SWV values which increased with age. The average SWV value of thyroid parenchyma in children with autoimmune thyroiditis was 3.7±1.2 (2.59-6.25) m/s which was statistically significantly greater than in healthy children (p=0.00). The cut-off value for elasticity with the highest diagnostic accuracy was 2.39 m/s; sensitivity and specificity were 97.4% and 100% respectively. There was no correlation between elasticity, thyroid function tests and autoantibody concentrations (p>0.05).

**Conclusion:**

SWE is a useful imaging method that can be used with routine ultrasonography in evaluation of the thyroid in children.

**What is already known on this topic?**

Shear wave elastography (SWE) provides real-time quantitative information about tissue elasticity by measuring and displaying local tissue elasticity. This non-invasive technique has the advantages of operator independence, reproducibility, high spatial resolution and quantitative evaluation without compression artifacts. There have been many studies about the advantages of SWE in adullts however studies in children are few.

**What this study adds?**

The aim of this study was to measure the elasticity of thyroid tissue in children and adolescents using SWE and to investigate the role of SWE in the diagnosis of autoimmune thyroiditis in childhood. Quantitative elastographic analysis evaluated by SWE in autoimmune thyroiditis patients (3.7±1.2 m/s) was significantly higher compared with normal pediatric patients (1.8±0.3 m/s) and the optimal cut-off value was 2.39 m/s.

## Introduction

Shear wave elastography (SWE) is a non-invasive method for measuring tissue elasticity whereby a quantitative estimate is provided of the elasticity of various soft tissues. It is a real time, quantitative, repeateable and user-independent imaging technique (1,2,3,4). With this technique, a short-time high-frequency acoustic repulsive force is applied using an ultrasonic probe, which causes small fluctuations in the tissues and the rate of advance of the formed waves through the tissue can be measured. The values of normal thyroid tissue elasticity in healthy adults are expressed as kilopascals (kPa) and shear wave velocity (SWV) in metres per second (m/s) in many studies, SWV of normal thyroid tissue of adults have been reported but few studies have been performed to evaluate the normal elasticity of thyroid tissue in children and adolescents (4,5,6).

Some pathologic conditions such as tumour and inflammation can change the normal tissue elasticity of thyroid. The elasticity measurement of thyroid tissue using SWE can be a useful, non-invasive test for the diagnosis of various thyroid diseases and this method has been performed successfully in the evaluation of thyroid tumours and autoimmune thyroiditis in adults (1,2,3).

Autoimmune thyroiditis is the most common thyroid pathology in childhood and adolescence (7,8). It is characterized by lymphocytic infiltration and fibrosis which may affect SWV. In studies performed in adults, SWV values were reported to be significantly higher in autoimmune thyroiditis than in normal thyroid parenchyma and its use as a dignostic method has been suggested. There are a few studies evaluating the elasticity of thyroid tissue with SWE in children with autoimmune thyroiditis (7,9,10,11). The aim of this study was to measure the elasticity of normal thyroid tissue with SWE in children and adolescents as well as to investigate the role of SWE in the diagnosis of autoimmune thyroiditis in childhood.

## Methods

### Healthy Control Group

One hundred and thirteen healthy children and adolescents of whom 66 were girls (58.4%) were evaluated with B-mode ultrasound and SWE. These children were recruited from patients referred for neck ultrasonography for non-thyroid pathologies (lymphadenopathy, thyroglossal cyst, brachial cyst, etc) between 4-14 years of age. Of these volunteers, those with a history of thyroid disease or a history of thyroid disease in their family were excluded. In all controls both left and right lobes were evaluated (226 lobes).

### Autoimmune Thyroiditis Patients

Fifty seven children and adolescents between 7-17 years of age of whom 45 (78.9%) were girls who were being followed up for a diagnosis of autoimmune thyroiditis in the pediatric endocrinology department of our hospital were evaluated with B-mode ultrasound, followed by SWE. The diagnosis of autoimmune thyroiditis was based on the presence of high levels of antithyroid antibodies including anti-thyroid peroxidase (TPOAb) and/or anti-thyroglobulin (TGAb), normal or low thyroid function assessed by measurement of thyroxine (T4) and thyroid stimulating hormone (TSH), together with assessment of heterogenity and hypoechogenity of thyroid parenchyma at ultrasound examination. Some of the patients were receiving antithyroid treatment during ultrasonography. All autoimmune thyroid patients had both lobes evaluated (114 lobes).

### Ultrasound Assessment

**Figure 1:**
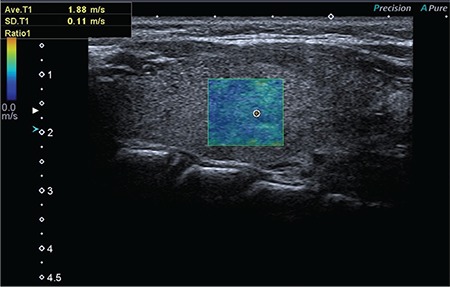
The evaluation of normal thyroid tissue with shear wave elastography

SWE measurements were performed using a linear transducer probe (7.5-10 MHz) with a Toshiba Applio 500 ultrasound machine (Toshiba, Japan). The evaluation of each thyroid gland was obtained with the children in the supine position and the neck in hyperextension, eased by positioning a pillow behind the neck. The measurements were performed in the longutidinal plane with sampling deeper than 1 cm and obtained during normal breathing. Measurement of SWV was made after checking the intensity of the signal. In healthy children, the region of interest (ROI) was 5×6 mm and the probe was placed perpendicular to homogenous parenchyma that did not include vessels or surrounding structures. The color-coded image showed soft tissue in blue and hard tissue in red. The quantitative evaluation of normal thyroid tissue in healthy children was performed by SWV (m/s). An average of five measurements were performed by an experienced pediatric radiologist and general radiologist in the healthy children at the same time in each of the two thyroid lobes (Figure 1). In healthy children, normal SWV values by age and averages were determined. Differences due to interobserver variability in the measurements were evaluated.

**Figure 2:**
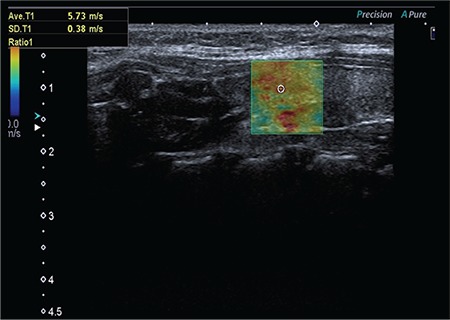
The evaluation of autoimmune thyroiditis with shear wave elastography

In autoimmune thyroiditis patients, SWE evaluation was done by one experienced pediatric radiologist, using the same technique (Figure 2). SWV values detected in patients with autoimmune thyroiditis were compared with SWV values of healthy children.

The mean examination time was 5.0±1.5 minutes (range 4-8 minutes).

### Ethical Aspects

This prospective study was approved by the local ethics committee (University of Health Sciences Bakırköy Dr. Sadi Konuk Training and Research Hospital Ethics Committee/2016/107). Written consent was obtained from patients and/or their parents.

### Statistical Analysis

This statistical analyses were performed with SPSS 22.0 software (IBM Inc., Chicago, IL, USA). Descriptive statistics of the data included mean, standard deviation, median, minimum, maximum, frequency and ratio values. The Kolmogorov-Smirnov test was used to analyze a range of variables. The Mann-Whitney U test, Wilcoxon and chi-square test were used in the analysis of quantitative data. The analysis of correlation was evaluated with the Spearman correlation test. Receiver-operating characteristic (ROC) curves were plotted for elasticity values and the optimum elasticity cut-off value that distinguished autoimmune thyroiditis from normal thyroid parenchyma was determined. Sensitivity, specificity, positive predictive value (PPV) and negative predictive value (NPV) were calculated.

## Results

**Figure 3:**
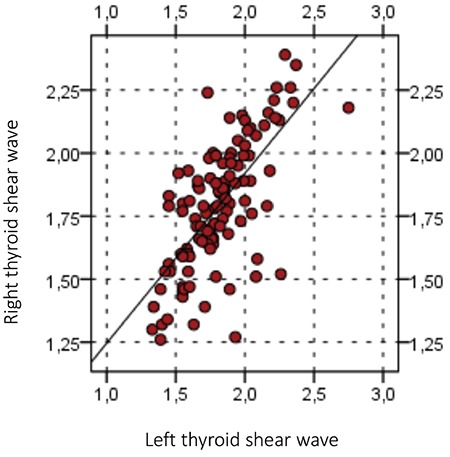
No significant differences are detected with shear wave velocity between the right and left thyroid lobes in healthy children

**Figure 4:**
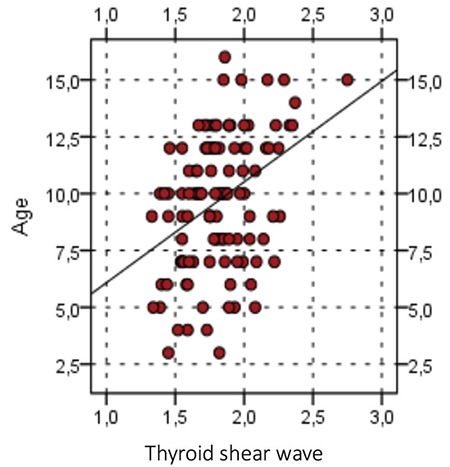
Positive correlation between age and shear wave velocity values

The mean ± standard deviation (SD) age of healthy children and adolescents was 9.7±2.9 years with a median of 10 year (range: 4-14 years). The mean ± SD SWV value of normal thyroid parenchyma was 1.8±0.3 m/s with a median of 1.85 m/s (range: 1.32-2.37 m/s). There was no significant difference between right and left thyroid lobes (p>0.05), nor between girls and boys (p>0.05) (Figure 3). There was a significant positive correlation between age and SWV values (r=0.390, p<0.001) (Figure 4).

**Figure 5:**
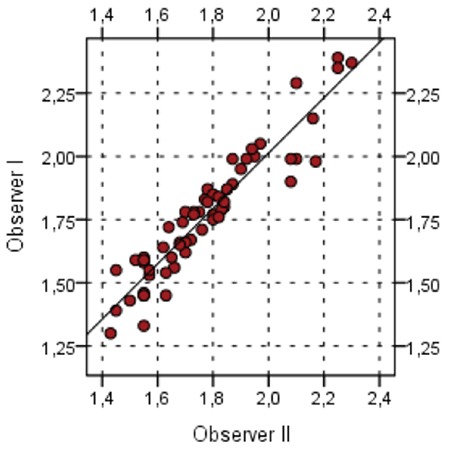
No significant differences were detected in intra-observer variability

Comparison of the two observers in the assessment of normal thyroid parenchyma SWV values was made. There was no significant difference for intra-observer results (p=0.624) and comparison showed a significant positive correlation (r=0.95, p<0.001) between the two (see Figure 5).

The mean age of children with autoimmune thyroiditis was 12.6±2.7 years with a median of 13 (7-17) years. The mean ± SD SWV values of thyroid parenchyma was 3.7±1.2 m/s with a median of 4.34 m/s in the patients (range: 2.59-6.25 m/s). There was no significant difference between right and left thyroid lobes (p>0.05) nor between girls and boys (p>0.05). In children with autoimmune thyroiditis, thyroid SWV values were significantly greater than those of healthy children (p≤0.05) (Table 1).

**Figure 6:**
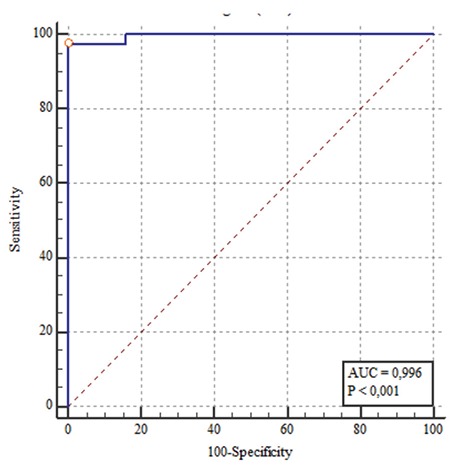
Receiver-operating charestics showing the optimal shear wave velocity cut-off value for autoimmune thyroiditis

ROC curves were plotted for elasticity values based on presence of autoimmune thyroiditis and area under the curves (AUC) was calculated. The maximum AUC for mean elasticity value of both lobes was 0.996 (AUC, 0.996; 95% confidence interval 0.968-1.0). The cut-off value with the highest diagnostic accuracy for elasticity value was 2.39 m/s; sensitivity, specificity, PPV and NPV were 97.4%, 100%, 100% and 99.1%, respectively (Figure 6).

SWV values and thyroid function tests showed no correlation with autoantibody concentrations in patients with autoimmune thyroiditis (p>0.05) (Table 2).

Seventeen of the patients were receiving antithyroid treatment during SWE. The mean treatment duration was five months (range 2-24 months). There was no significant correlation between SWV values and antithyroid treatment duration (p>0.05).

## Discussion

Many studies on evaluation of normal thyroid tissue and its pathologies by SWE have been reported in adult populations (5,6,12,13,14,15). However there are few studies using this imaging method for the assessment of thyroid tissue in children. In this study we standardized a protocol for normal thyroid SWV measurements with regard to frequency, measurement depth and position, size of ROI and acquisition number in a pediatric population. We used high linear frequency probes and measured the SWV at a depth of more than one centimetre below the front edge of each thyroid lobe. Children who were able to cooperate were asked to hold their breath for a short time. In younger children, the study was performed with free-breathing. The operator applied similar amounths of transducer pressure only necessary to create a gray scale image thus avoiding preload. ROI was placed perpendicular to a homogeneous parenchyma that did not include vessels or surrounding structures. This results in the thyroid tissue appearing perfectly homogeneous with a colour code corresponding to a soft tissue (blue colour code). The ROI size was small and it allowed for more accurate measurements. The size of ROI was determined as 5x6 mm, similar to that used for adults. In our study, five valid SWVs for each thyroid lobe were obtained because most children could not tolerate a prolonged examination. The mean examination time was 5±1.5 minutes (range 4-8 minutes) in our study.

The SWE measurement technique for thyroid in adults has been reported by the World Federation for Ultrasound in Medicine and Biology in 2017 (9). The recommended measurement technique for adults is similar to the technique we used in children. However, there were some notable differences in technique. Young children cannot cooperate, thus cannot be required to hold their breath during SWE. The ROI used is standardized as 5x6 mm because of the small size of the thyroid in children. In addition, the measurement depth recommended for adults is 4-5 centimetres but SWE measurement of thyroid tissue in children, due to age dependent thyroid size, was evaluated as any depth above one centimetre. The measurements of thyroid tissue in adults is recommended to be from 5-6 different areas for each thyroid lobe. Sporea et al (13) reported that SWE of the thyroid is feasible with linear and convex probes and five measurements in every lobe are sufficient for an accurate assessment of thyroid tissue. Sporea et al (13) reported no significant difference between five or 10 measurements for thyroid stiffness in adults with acoustic radiation force impulse elastography. Vlad et al (14) performed three measurements on each thyroid lobe and calculated a mean elasticity in healthy adults. In our study, five SWV measurements were made in the children and the mean values were calculated as previously described by Ceyhan Bilgici et al (10).

We found no significant intra-observer variability for SWV measurements of thyroid tissue. Bhatia et al (16) reported that inter- and intra-operator reproducibility in SWE is acceptable with correlations ranging from 0.78 to 0.85 for intra-observer variability and between 0.97 and 0.98 for inter-observer variability. Bilgici et al (10) only assessed inter-observer variability and they found a value of 0.70 for the right lobe and 0.69 for the left lobe.

SWV values for normal adult thyroid tissue have been reported in various studies. These values were obtained from healthy control groups formed during SWE evaluation of diffuse thyroid diseases and thyroid nodules. Arda et al (4) reported, in an adult populations, a mean elasticity value of 10.97+-3.1 kPa (approximately 1.89 m/s) for the thyroid. Fukuhara et al (17) found that the SWV value for normal thyroid tissue was 1.60±0.18 m/s in adults. In healthy adults, Friedrich-Rust et al (6) reported a mean SWV value of 1.98 m/s, while Hekimoglu et al (15) reported an SWV value of 1.63±0.12 m/s and that the range of SWV was between 1.59 and 1.98 m/s in adults.

In our study, the mean SWV values of normal thyroid parenchyma was 1.82±0.3 m/s with a range of between 1.32 and 2.37 m/s in healthy children. There was no significant difference between girls and boys. However, there was a significant positive correlation between age and SWV values, which increased with age. Studies have shown that changes in thyroid function occur with age and that the size of the thyroid gland shows a significant increase with puberty. Therefore, the increase in SWV values with age appear to follow, in the first years the elevation of TSH and the decrease in free T4 and free T3 hormones and the increase of thyroid volume with age (18,19). Ceyhan Bilgici et al (10), who conducted the first studies in this area in children, reported a mean SWE value of 1.22±0.20 m/s for the thyroid gland at a mean age 10.5±3.1 years. In addition, in contrast to our study, they did not find any correlation between age, thyroid gland volume and body mass index. Arioz Habibi et al (20) reported that SWV values of the thyroid gland were significantly higher in the 13 to 17 years age group and that there was a significant positive correlation between age and SWV values. These authors explained this finding as a function of age elasticity values of the thyroid which do not show a significant difference up to 12 years of age. The low SWE values may be explained by differences in age groups and thyroid hormone differences. In our study, a significant number of patients were at late childhood and adolescent ages.

The number of studies on the use of SWE in diffuse thyroid pathologies in adults is limited. Most of these studies concern chronic autoimmune thyroiditis (CAT). This is the subgroup of autoimmune thyroiditis which causes fibrosis in the thyroid gland. Autoimmune thyroiditis is also the most common thyroid pathology in childhood and adolescence (7). There are few studies evaluating the elasticity of thyroid tissue with SWE in children with autoimmune thyroiditis. Autoimmune thyroid diseases in children are usually diagnosed on the basis of clinical and laboratory findings, supported by ultrasound. The pathological features of autoimmune thyroiditis are interstitial infiltration by lymphocytes and a variable degree of fibrosis in tissue. It is thought that fibrosis leads to high SWV values as stiffness of a tissue is correlated with increased values of SWV.

Fukuhara et al (17) found that the SWV value in CAT (2.47±0.57 m/s) was significantly higher than in healthy adults (1.59±0.41 m/s) and that the SWV cut-off  value was 1.96 m/s. Hekimoglu et al (15) reported a mean SWV value of 1.63±0.12 m/s in normal adults and 2.56±0.30 m/s in adults with CAT. They found that the optimal cut-off  value for CAT prediction was 2.42 m/s (77% sensitivity, 71% specificity, 92% PPV, 81% NPV and 87% accuracy).

Sporea et al (21) found significant difference in SWV in autoimmune thyroid disease with a value of 2.07±0.44 m/s. They found 2.68±0.50 m/s in Graves disease and 2.34±0.61 m/s in CAT in adults. They reported a cut-off  value >2.53 m/s for differentition between normal thyroid tissue and diffuse thyroid diseases with a PPV >90%. Kim et al (22) found a cut-off  value of 27.6 kPa (about 3.96 m/s), with a sensitivity of 40.9% and specificity of 82.9%. Vlad et al (14) stated that SWE may predict the presence of autoimmune thyroid disease. They found the best cut-off  value for predicting thyroid pathology by SWE as 22.3 kPa (about 3.20 m/s) with a sensitivity of 59.6% and a specificity of 76.9%. Yucel et al (11) reported an SWV value of 1.67±0.63 m/s in Hashimoto patients, a significantly higher value compared to healthy children. They reported an optimal cut-off  value of 1.41 m/s with 73.1% sensitivity, 80.8% specificity, 79.2% PPV and 75% NPV. Kandemirli et al (12) determined 14.9 kPa in pediatric patients with Hashimoto’s thyroiditis and reported significantly higher elasticity values than healthy subjects. The elasticity value cut-off  with the highest diagnostic accuracy was 12.3 kPa and 1.968 m/s; sensitivity, specifity, PPV, NPV, and diagnostic accuracy of this cut-off  were 86.4%, 96.3%, 98.1%, 76.5%, and 89.5%, respectively.

Our data is similar those published by Vlad et al (14). We found that normal thyroid parenchyma appears homogeneous, with low elasticity colored in blue, and autoimmune thyroiditis appears heterogeneous with areas of yellow and red scattered among the blue. The average SWV value of thyroid parenchyma in pediatric autoimmune thyroiditis was 3.7±1.2 m/s and ranged from 2.59 to 6.25 m/s. In our study, the mean SWV measurement was higher than that of adults and also higher than values reported by Yucel et al (11) and Kandemirli et al (12). Fukuhara et al (17) found that SWV values are significantly affected by fibrosis but seldom by cellular density. This finding suggests that fibrosis may have an effect in the pathology of autoimmune thyroid diseases in children. Kandemirli et al (12) reported that SWV values increased as the degree of fibrosis increased in CAT.

In our study, when the correlations between SWV values and autoantibody levels and thyroid function tests were analyzed in patients autoimmune thyroiditis, no correlation was found. However, Magri et al (23) reported a positive correlation between tissue stiffness and serum TPOAb and concluded that TPOAb values of the patients could affect SWV measurements. In contrast, Liu et al (24) found that thyroid stiffness was weakly related to TSH and TGAb and was not correlated with T3, T4 or TPOAb. Kandemirli et al (12) reported a moderate but significant correlation between elasticity values and TPOAb but no significant correlation between SWV values and TGAb levels. They explained this by suggesting that the presence of TPOAb might be characteristic of a late adaptive immune response whereas TGAb might reflect an early immune response. Yucel et al (11) found a correlation between TPOAb values and SWV values in patients but no correlation between SWV and either TGAb or thyroid function test results. Thyroid elasticity cannot currently be used to predict thyroid functions.

Ruchala et al (25) concluded that SWE might be useful in the diagnosis and differentiation between various types of thyroiditis but was not the optimal tool to differentiate Graves disease and CAT. Liu et al (24) found that SWE is helpful for distinguishing Graves disease from subacute thyroiditis but that it is unsuitable for differentiating CAT and Graves disease (24). All types of thyroiditis are characterised by increased thyroid stiffness and in CAT patients the degree of stiffness increases with fibrosis progression. Studies have shown that medical treatment in the presence of autoimmune thyroiditis has no effect on the elasticity of the thyroid tissue in adults (26). Some of our patients were using medical treatment during SWE. There was no significant correlation between elasticity values and antithyroid treatment with treatment duration in our study.

### Study Limitations

The first of our limitations was that the mean age of our autoimmune thyroiditis cases was higher than our normal control cases. The reason is that these pathologies are more common in late childhood and adolescence. The second limitation was that only autoimmune thyroiditis patients were evaluated in the study. There is a need for studies on SWV measurements to demonstrate how this measurement is affected in other diffuse thyroid pathologies in children.

## Conclusion

In this study, which evaluated the elasticity values of normal thyroid tissue, the measured values are presented. We also showed that the SWV values increased with age. In addition, it was demonstrated that SWV values in autoimmune thyroiditis patients were significantly higher compared to those of healthy children. There is a need for further studies to establish normal values for each age group. Also, further studies with larger series of children and adolescents are needed to compare the elasticity values of normal and pathologic tissues, such as diffuse thyroid diseases, thyroid nodules, etc, to determine the diagnostic role of this imaging technique in children. SWE is a useful imaging method complementing routine ultrasonography examination in pediatric patients in whom a diagnosis of autoimmune thyroid disease is considered.

## Figures and Tables

**Table 1:**
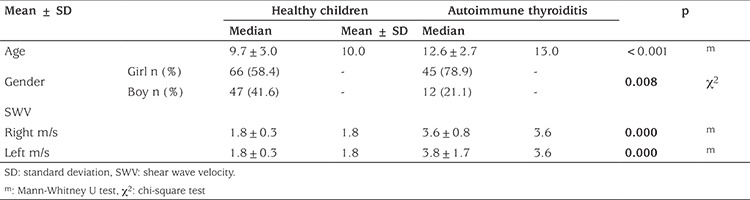
Comparison of thyroid shear wave velocity values in healthy children and children with autoimmune thyroiditis

**Table 2:**
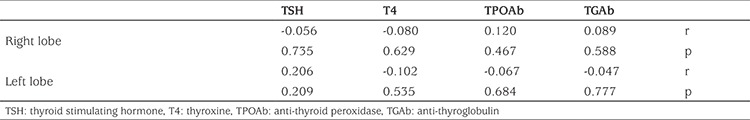
Correlation between thyroid function tests and autoantibody titers and thyroid tissue elasticity values
